# Sources of variation in estimates of Duchenne and Becker muscular dystrophy prevalence in the United States

**DOI:** 10.1186/s13023-023-02662-0

**Published:** 2023-03-22

**Authors:** Nedra Whitehead, Stephen W. Erickson, Bo Cai, Suzanne McDermott, Holly Peay, James F. Howard, Lijing Ouyang, the Muscular Dystrophy Surveillance, Tracking and Research Network

**Affiliations:** 1 Social, Statistical, and Environmental Sciences, RTI International, 2987 Clairmont Road NE, Atlanta, GA USA; 2 Social, Statistical, and Environmental Sciences, RTI International, Research Triangle Park, NC USA; 3 Arnold School of Public Health, University of South Carolina, Columbia, SC USA; 4 Department of Environmental, Occupational, and Geospatial Health Sciences, City University of New York Graduate School of Public Health and Health Policy, New York, NY USA; 5 Department of Neurology, The University of North Carolina, Chapel Hill, NC USA; 6 National Center on Birth Defects and Developmental Disabilities, Centers for Disease Control and Prevention, Atlanta, GA USA

**Keywords:** Epidemiology, Public health surveillance, Epidemiological monitoring, Epidemiologic methods, Muscular dystrophy Duchenne, Muscular dystrophy Becker, Prevalence

## Abstract

**Background:**

Direct estimates of rare disease prevalence from public health surveillance may only be available in a few catchment areas. Understanding variation among observed prevalence can inform estimates of prevalence in other locations. The Muscular Dystrophy Surveillance, Tracking, and Research Network (MD STAR*net*) conducts population-based surveillance of major muscular dystrophies in selected areas of the United States. We identified sources of variation in prevalence estimates of Duchenne and Becker muscular dystrophy (DBMD) within MD STAR*net* from published literature and a survey of MD STAR*net* investigators, then developed a logic model of the relationships between the sources of variation and estimated prevalence.

**Results:**

The 17 identified sources of variability fell into four categories: (1) inherent in surveillance systems, (2) particular to rare diseases, (3) particular to medical-records-based surveillance, and (4) resulting from extrapolation. For the sources of uncertainty measured by MD STAR*net,* we estimated each source’s contribution to the total variance in DBMD prevalence. Based on the logic model we fit a multivariable Poisson regression model to 96 age–site–race/ethnicity strata. Age accounted for 74% of the variation between strata, surveillance site for 6%, race/ethnicity for 3%, and 17% remained unexplained.

**Conclusion:**

Variation in estimates derived from a non-random sample of states or counties may not be explained by demographic differences alone. Applying these estimates to other populations requires caution.

**Supplementary Information:**

The online version contains supplementary material available at 10.1186/s13023-023-02662-0.

## Background

Public health surveillance, defined as the "systematic and continuous collection, analysis, and interpretation of data" [1] is foundational to public health practice [2]. Public health surveillance provides accurate, representative information on the occurrence of a disease in the population from which the data is collected but is not usually designed to be generalizable to other populations. Resources and logistics may limit surveillance programs to a few catchment areas that may not be representative of the entire population. In the absence of other data, prevalence and other epidemiologic measures from these few catchment areas are often generalized to the population, which is valid only if the epidemiology of the disease of interest is consistent across the population.

Significant variation in epidemiologic measures among catchment areas suggests the underlying epidemiology of the disease differs among geographic areas. However, rare diseases are vulnerable to random fluctuation in prevalence estimates, which can be difficult to distinguish from true differences among populations. Structured uncertainty analysis can be an important tool for assessing such differences. Taruscio and Mantovani recently demonstrated the value of uncertainty analysis to identify gaps in our knowledge of the epidemiology of rare diseases and assess their impact [3]. They categorize the sources of uncertainty into epistemic (uncertainty due to lack of knowledge), sampling uncertainty (uncertainty associated with data and disparate methods), and variability (uncertainty due to heterogeneity within a population).

The Muscular Dystrophy Surveillance, Tracking and Research Network (MD STAR*net*), which conducts population-based surveillance of muscular dystrophies in selected areas of the US, is the sole source of population-based prevalence estimates in the country [4, 5]. The 2007 MD STAR*net* estimated prevalence of Duchenne/Becker muscular dystrophy (DBMD) among males age 5 to 24 was 1.47 cases per 10,000 males (calculated from data in the article) [6, 7]. The range among the four individual catchment areas was 1.3 to 1.8 cases per 10,000 males ages 5 to 24 years, a variance of 12% [6]. Among the three catchment areas with estimates for 2007 and 2014–2019, the same catchment areas had higher prevalence in both time periods, indicating that the differences between catchment areas are likely not random (Personal communication, Suzanne McDermott, DBMD Ascertainment Progress Presented: Fall 2017 MD STARnet Principal Investigators Meeting. Atlanta, GA, 2017).

Variation across catchment areas could be due to true differences in the population frequency of pathogenic alleles of the dystrophin gene; the population distribution of sex, age or ancestry; or migration among individuals with DBMD. It could also be due to random or systematic error. Our aim was to understand what factors explain the observed differences in DBMD prevalence among catchment areas and the implications for the generalizability of the prevalence estimates. Our analysis examined sources of sampling uncertainty and population variability. If population demographics or regional differences in diagnosis or surveillance practices explain the variation among catchment areas, adjustment for these differences would allow MD STAR*net* estimates to be extrapolated to the broader U.S. population. Unexplained variation between catchment areas indicates that MD STAR*net* prevalence estimates may not be an accurate estimate of DBMD prevalence in the broader U.S. population.

## Results

### Literature review and investigator survey

After abstract and title review, we identified 52 unique citations, of which 12 advanced to full text review (Additional file 1: Fig. S2, Additional file 2). We included findings from five articles, from which we identified 12 potential sources of variation (Table 1) [8–12]. None of the minor discrepancies in abstraction required adjudication. Most information on sources of variation was in surveillance or registry methodological articles. These articles examined rare disease cluster identification [8], drug registries for treatments of lysosomal storage disorders [9], a cancer registry [11], and surveillance based on multiple data sources [12]. The fifth article was an epidemiological report from a registry of arthritis, musculoskeletal and skin diseases [10].

**Table 1:** Sources of variation in estimating the national prevalence of muscular dystrophies

Source of Variation	Identified from	
In All Surveillance	Literature	Survey
Unidentified or unavailable data sources	x	x
Unidentified cases at known data sources		x
Misclassified disease status		x
Migration into and out of surveillance system	x	
Time period for case capture	x	x
Time between diagnosis and ascertainment		x
Regional differences in disease incidence	x	
Unreliable, non-specific coding in screening databases	x	x
Migration into and out of surveillance region	x	x
Demographic changes due to rapid population change	x	x
Specific to Rare Disease Surveillance		
Unstable estimates due to small number of cases	x	
Misclassification of muscular dystrophy type	x	x
Specific to Medical Records-Based Surveillance		
Lack of standardized data in medical records	x	
Underreported and incomplete data in medical records	x	x
Number and proportion of treatment centers within the study area		x
Specific to Extrapolation to National Estimates		
Differences between study population and national population		x
Differential ascertainment between areas or groups of patients	x	

Twenty investigators from six sites completed our survey on sources and magnitude of bias in MD STAR*net*. The investigators included six analysts, four abstractors, three clinicians, three study coordinators, two data managers, and two people with unspecified roles. The survey identified 12 sources of variation, five of which had not been identified by the literature review (Table 1). The average investigator estimate of bias in DMD prevalence from a given source ranged from 5% (for residents obtaining care outside the study region and demographic changes in the population) to 12% (for differences between the MD STAR*net* and the U.S population) (Additional file 3: Table S1).

In total, we identified 17 sources of variation in national estimates from the literature review or the investigator survey (Table 1). We grouped the sources of variation into four categories comprising sources of variation that are:

1. Inherent to all surveillance systems, including case ascertainment, misclassification of disease status, and migration;
2. Specific to rare disease surveillance, including small case numbers, regional differences in incidence, the relatively large impact of a few misclassified cases, and biases in care-seeking behaviors and diagnostic practices;
3. Specific to medical records-based surveillance, including lack of standardization and incomplete data; and
4. Due to extrapolation from local to national estimates, including differences between the local and national populations.

### Sources and magnitude of variation

The expanded MD STAR*net* data set included 720 cases from a surveilled male population of 8 million (Table 2). Of these cases, 249 (34%) were identified in Arizona, 193 (27%) in Colorado, 152 (21%) in Iowa, and 126 (17%) in western New York. The cases were mostly non-Hispanic and white (67%). The racial and ethnic distribution of the cases was similar to that of the surveilled populations, although individuals of Black or Other race were slightly underrepresented among the cases.

**Table 2:** Sample and Population Characteristics, MD STAR*net* Expanded Surveillance Pilot, 2007–2011

	DBMD cases		Surveilled population		US Male Standard Population	
	Number	Percent	Number	Percent	Number	Percent
Male	720	100	8,037,535	100.0	152,082,993	100.0
Age (years)						
Under 5	61	8.5	553,842	6.9	10,312,641	6.8
5 to 9	116	16.1	565,909	7.0	10,380,281	6.8
10 to 14	131	18.2	560,581	7.0	10,578,235	7.0
15 to 19	139	19.3	592,868	7.4	11,278,027	7.4
20 to 24	101	14.0	585,417	7.3	11,072,538	7.3
25 +	172	23.9	5,178,918	64.4	98,461,271	64.7
Race/Ethnicity						
Black	19	2.6	347,436	4.3	18,116,746	11.9
Hispanic	146	20.3	1,602,343	19.9	25,749,686	16.9
Other^1^	69	9.6	481,365	6.0	11,151,601	7.3
White	486	67.5	5,606,391	69.8	97,064,960	63.8
State						
Arizona	249	34.6	3,175,823	39.5	NA	
Colorado	193	26.8	2,520,662	31.4		
Iowa	152	21.1	1,508,319	18.8		
New York	126	17.5	832,731	10.4		

^1^Includes any race other than Black, Hispanic, or White, including multiple races and missing

DBMD, Duchenne/Becker Muscular Dystrophy; US, United States

Age and ethnicity distributions were significantly associated with prevalence. Age group explained the majority of the variability between strata, accounting for 74% of the deviance in the model. However, the similarity of unadjusted, standardized, and adjusted prevalence estimates indicates that population differences in age and ethnicity or differences in the surveillance process account for very little of the variation between catchment areas (Table 3). Catchment area accounted for the second largest proportion of the variability between strata, 6% of the total variance (Table 4). Arizona was the reference site due to alphabetical coding order. Prevalance in Colorado and Iowa did not differ significantly from those in Arizona (Table 5). However, the prevalence in the New York catchment area was twice that of Arizona (Prevalence Ratio. 2.2, *p* < 0.001). Seventeen percent of the variation in prevalence across strata remained unexplained after controlling for the demographic and process factors in the model.

**Table 3:** Unadjusted, Standardized and Adjusted Duchenne and Becker Muscular Dystrophy Prevalence by Participant Characteristics, MD STAR*net* Expanded Surveillance Pilot, 2007–2011

	Unadjusted		Standardized^1^		Adjusted^2^	
	Prevalence^3^	95% CI	Prevalence^3^	95% CI	Prevalence^3^	95% CI
All US males	8.96	8.33, 9.64	8.68	8.03, 9.38	8.64	7.97, 9.33
Age (years)						
Under 5	11.01	8.58, 14.15	10.17	7.77, 13.08	10.73	8.04, 13.59
5 to 9	20.50	17.09, 24.58	20.59	16.81, 24.97	19.93	16.26,23.80
10 to 14	23.37	19.70, 27.73	22.77	18.82, 27.31	22.69	18.72, 26.84
15 to 19	23.45	19.86, 27.68	23.59	19.51, 28.27	22.65	18.80,26.65
20 to 24	17.25	14.20, 20.96	15.95	12.95, 19.45	16.61	13.38,20.04
25 +	3.32	2.86, 3.86	3.23	2.76, 3.77	3.23	2.74, 3.74
Race/Ethnicity						
Black	5.47	3.50, 8.54	5.49	3.31, 8.58	5.47	3.14, 8.10
Hispanic	9.11	7.75, 10.71	8.79	7.42, 10.34	8.73	7.29, 10.22
Other^4^	14.33	11.33, 18.14	13.49	10.48, 17.09	13.11	10.05,16.37
White	8.67	7.93, 9.47	8.70	7.94, 9.51	8.70	7.91, 9.51
State						
Arizona	7.84	6.93, 8.88	7.22	6.29, 8.25	7.46	6.52, 9.26
Colorado	7.66	6.65, 8.82	7.64	6.51, 8.92	7.36	6.19, 9.24
Iowa	10.08	8.60, 11.81	9.62	7.82, 11.71	9.89	7.89, 12.46
New York	15.13	12.71, 18.01	13.46	10.90, 16.45	14.30	11.53,18.97

MD STAR*net*, Muscular Dystrophy Surveillance, Research and Tracking Network; US, United States; CI, confidence interval

^1^Standardized to US male population by age and race/ethnicity

^2^Adjusted by age, race/ethnicity, site, number of reporting sources, and proportion of cases seen at a neuromuscular clinic. Based on multivariable Poisson model, with confidence intervals obtained from 100,000 random simulations

^3^Per 100,000 individuals

^4^Includes any race other than Black, Hispanic, or White, including multiple races and missing

**Table 4:** Analysis of deviance, MD STAR*net* Expanded Surveillance Pilot, 2007–2011

Variable	Degrees of freedom	Deviance	Percent of deviance
Age	5	527.0	73.9%
State	3	41.5	5.8%
Race/ethnicity	3	19.8	2.8%
Proportion treated at MD clinic^1^	1	3.3	0.4%
Average number of ascertainment sources^2^	1	0.1	< 0.1%
Proportion diagnosed by genetic testing^3^	1	0.0	< 0.1%
Residuals	81	121.4	17.0%

MD STAR*net*, Muscular Dystrophy Surveillance, Research and Tracking Network

^1^Proportion of patients within stratum who were treated at a neuromuscular clinic

^2^Average number of the number reporting sources at which each patient in stratum was identified

^3^Proportion of cases diagnosed by genetic testing in the index case or a family member

**Table 5:** Association of Population Characteristics with Prevalence of Duchenne/Becker Muscular Dystrophy^1^, MD STAR*net* Expanded Surveillance Pilot, 2007–2011

	Prevalence Rate Ratio	95% Confidence Interval	*P*-value
Age (years)			
Under 5	0.504	0.366–0.686	< 0.001
5 to 9	0.901	0.690–1.174	0.427
10 to 14	Ref.		
15 to 19	0.994	0.775–1.275	0.959
20 to 24	0.732	0.558–0.957	0.020
25 +	0.170	0.115–0.250	< 0.001
State			
Arizona	Ref.		
Colorado	1.164	0.786–1.721	0.444
Iowa	1.368	0.930–2.001	0.086
New York	2.164	1.620–2.875	< 0.001
Race/Ethnicity			
Black	0.501	0.301–0.785	0.004
Hispanic	0.882	0.716–1.081	0.233
Other	1.424	1.077–1.856	0.009
White	Ref.		
Average number of ascertainment sources^2^	1.095	0.735–1.629	0.650
Proportion diagnosed by genetic testing^3^	1.009	0.533–1.915	0.993
Proportion treated at MD clinic^14^	2.696	0.905–8.164	0.073

^1^The dependent variable was number of Duchenne and Becker muscular dystrophy cases, with the logarithm of stratum population used as an offset variable

^2^Average number of reporting sources for each patient in stratum

^3^Proportion of cases diagnosed by genetic testing in the index case or a family member

^4^Proportion of patients within stratum that were treated at a muscular dystrophy clinic

## Discussion

Our primary goal was to determine whether adjusting for sources of variability in site-specific prevalence estimates would reduce differences among catchment areas, increasing confidence that findings are generalizable beyond the areas included within the surveillance system. Unfortunately, adjusting for known and potential sources of variability by standardization or multivariate modeling did not substantially reduce between-site differences. Surveillance site accounted for 6% of the deviance between prevalence rates, and 17% of the deviance was unexplained after adjusting for age, race/ethnicity, and ascertainment details. The large proportion (74%) of the deviance explained by age group is expected given the natural history of DBMD. In this progressive disorder, prevalence is low in children younger than the usual age of diagnosis (approximately 5 years) and highest among children age 5–19 years, when most affected boys have been diagnosed and mortality is still low. The prevalence declines among adults age 20 years and older, when mortality increases.

Our analysis complements the article by Taruscio and Mantovani ^3^ by providing an example of a structured analysis to evaluate the uncertainty in prevalence estimates of rare diseases. We experienced several challenges in analyzing the sources of variability. Population level data on potential sources of variation such as the number of unsurveilled health care providers within a catchment area was unavailable. We could not evaluate how well our proxy measures, the mean number of sources at which cases were ascertained and the proportion of cases seen at a neuromuscular clinic, estimated the completeness of coverage of health care facilities treating muscular dystrophy for each stratum. Socioeconomic status was unavailable at the case level. The limited data on potential sources of variability and the relatively small number of strata limited our ability to explain the sources and magnitude of variation in DBMD prevalence rates.

Our analysis is strengthened by factors that reduce process variability in case ascertainment. MD STAR*net* sites use a standard protocol [4]. Cases are actively sought using multiple data sources, and identifying information allows duplicate cases to be identified and consolidated. For the pilot, case eligibility was reviewed by a local clinician experienced in treating muscular dystrophy cases, with additional review of uncertain cases by a committee of clinicians [4, 13].

Our findings suggest that the estimated prevalence of muscular dystrophy may be dependent on which sites are included in MD STAR*net*. More generally, they suggest that estimates derived from a non-random sample of states or counties cannot be assumed to represent national rates. Although not all the factors that impact MD STAR*net* estimates are generalizable to other surveillance systems, our study illustrates a valuable approach for evaluating the sources and impact of uncertainty that is applicable to rare disease surveillance systems generally. This analysis provides an example of one methodology for such an evaluation. The Poisson model we used provided estimates of the magnitude and relative contribution of each potential source of variability of DBMD prevalence across demographic strata within the limitations of our data.

## Conclusions

Estimating sources of variability in the extrapolation of the prevalence of DBMD from a local to a national scale requires attention to surveillance methodology, the characteristics of the condition under surveillance, and differences and similarities between the local and national populations. In this study, 17% of the variation was not explained by the model.

## Methods

Our objectives were to identify sources of variation in MD STAR*net* prevalence estimates between sites and to estimate the magnitude of the total variation in DBMD prevalence estimates and the relative contribution of each source of variation.

### Sources of variation

**Fig. 1:**
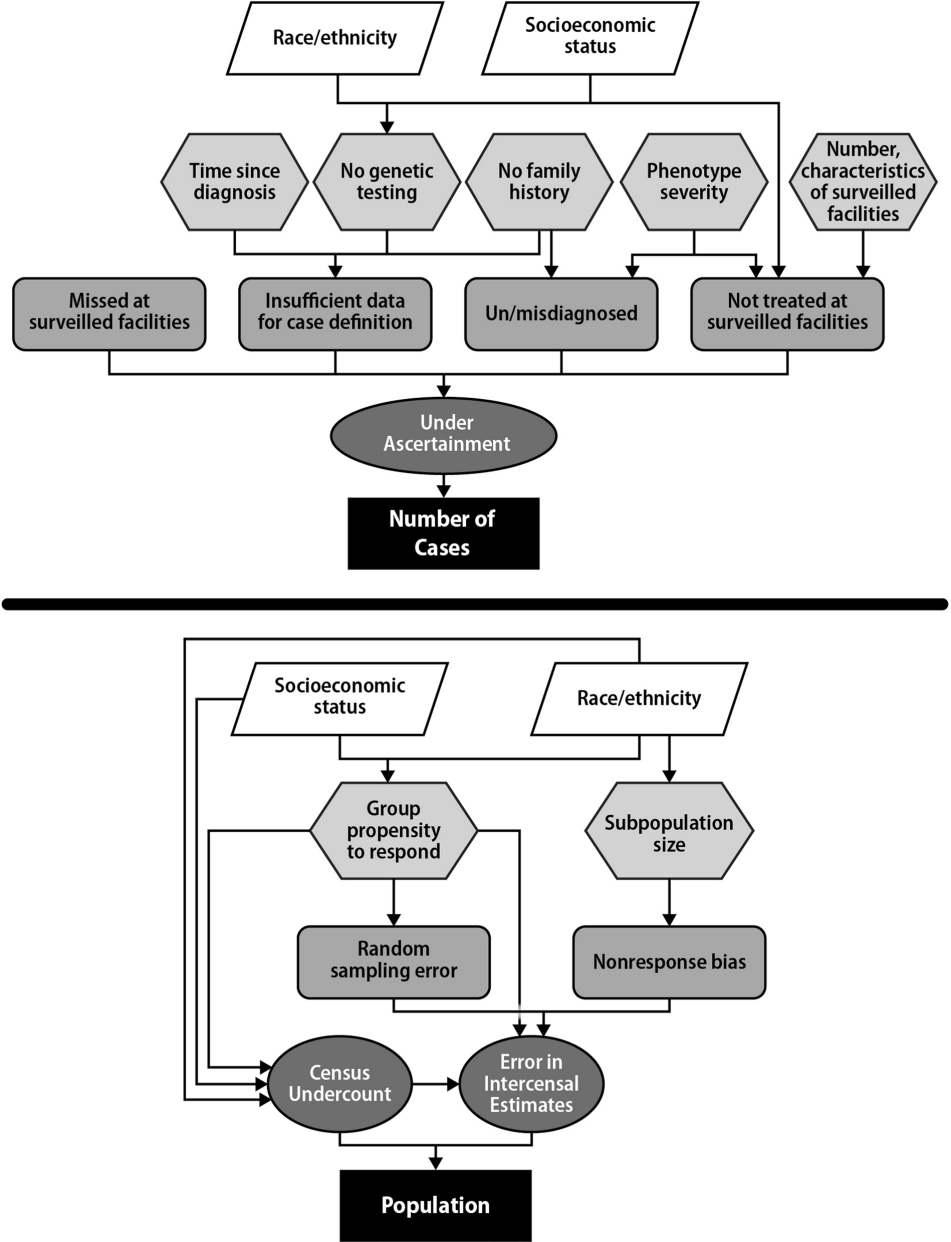
Sources of variation in prevalence estimates - conceptual model

We identified potential sources of variation in prevalence estimates from the scientific literature and expert opinion. We synthesized the findings into a theoretical model of how the sources contributed to potential bias in generalizing the estimates to the US population (Fig. 1).

*Literature review.* Two analysts independently searched PubMed and Google Scholar and reviewed the retrieved citations for eligibility. Our original criteria for inclusion were methodological studies of the types, sources, or magnitude of bias in surveillance or research studies. PubMed and Google Scholar were chosen because they were available to both analysts and were expected to capture most articles on public health surveillance methods. The search terms included surveillance, rare disease, prevalence, error, limitations, uncertainty, epidemiology, estimation, MD STAR*net*, muscular dystrophy, prevalence, US Census, and variations of these terms. Details on the search strategies are provided in the Additional file 4. The last search was conducted on November 3, 2016 and included all articles published prior to that date. The search was not updated after the final logic model was constructed.

We adhered to a rigorous search methodology to the extent possible but deviated from a full systematic review methodology in two regards. First, we could not develop a complete, deduplicated count of identified citations because Google Scholar results cannot be exported, making it impossible to identify duplicates. Second, we found very few studies that met our pre-determined eligibility criteria of being designed explicitly to study the sources or magnitude of bias in surveillance systems. Instead, information on sources of bias was more commonly found in reports about surveillance or research study design. We therefore include articles that discussed possible sources of bias in their surveillance system or data even if they did not estimate the magnitude of the bias. The placement of the information within the article and the depth of detail varied greatly among studies. This variability made the use of structured abstraction or a data extraction tool impossible. Instead, relevant information was manually extracted into Word.

Both analysts reviewed the combined list of eligible citations and classified each as included or excluded. Included articles were abstracted by both analysts independently and reviewed for discrepancies.

*Investigator survey.* We surveyed MD STAR*net* investigators to explore their experiences and perceptions of different sources of variation that may affect MD STAR*net* prevalence estimates, and the approximate magnitude of bias that may be introduced by each source (Additional file 5: Fig. S1). Due to the small number of eligible sites, instead of formally piloting the survey, it was reviewed by North Carolina investigators who did not participate in developing the survey. We emailed the link to the Survey Gizmo [14] survey to the principal investigators of six sites (Colorado, Iowa, western New York, central North Carolina, South Carolina, and Utah) funded from 2014 to 2019 and asked them to distribute it to the MD STAR*net* investigators at their site. Because staff roles and responsibilities vary across MD STARnet sites, we relied on the principal investigators to distribute the survey to appropriate site colleagues. The survey was anonymous; investigators who responded online could not be identified or linked to a specific site, and a formal response rate could not be calculated. There was at least one response from all sites. Four sites submitted responses through the link, and two sites submitted aggregate responses for their site by email. The institutional review board (IRB) at RTI International, employer of the primary analysts, determined the survey was program evaluation, not human subjects research as defined by 45 CFR 46.102. Due to the small sample size and the aggregate responses obtained from two sites, all data were analyzed descriptively.

### MD STAR*net* data

The analytic data were from MD STAR*net*’s pilot expanded muscular dystrophy surveillance (EMDS) [4]. Four geographically defined surveillance sites (Arizona, Colorado, Iowa, and 12 counties in western New York) conducted retrospective active surveillance of nine muscular dystrophies (MD) (Duchenne, Becker, congenital, distal, Emery-Dreifuss, facioscapulohumeral, limb-girdle, and oculopharyngeal MD, MD not otherwise specified, and myotonic dystrophy) from 2011 to 2014. All four sites had authority to conduct public health surveillance by the legal authority of their state department of health and/or institutional review board approval or exemption [4]. Informed consent was waived because the project was public health surveillance. Trained medical coders reviewed electronic or paper medical records of eligible cases to abstract information about signs and symptoms, diagnostic tests, treatment and follow-up care. Eligible individuals had evidence of a physician’s diagnosis of a specific MD type within their medical record, resided within a MD STAR*net* catchment area, and had at least one healthcare encounter from 2007 to 2011 inclusive [4]. Case ascertainment sources varied between sites but included physician and other provider medical records, hospital records, vital statistics, and administrative data. Cases were ascertained using International Classification of Diseases, Ninth Revision, Clinical Modification codes (359.0: congenital hereditary MD, 359.1: hereditary progressive MD, 359.21: myotonic dystrophy) in medical and administrative records and International Classification of Diseases, Tenth Revision mortality codes (G71.0: MD, G71.1: myotonic dystrophy) in death certificates. At each site, a clinician who treated patients with muscular dystrophy reviewed the abstracted case notes and decided if the MD type specified was consistent with standard diagnostic practice. If the diagnosis was in question, a panel of 5 neuromuscular experts made the final determination about MD type. The muscular dystrophies differ in inheritance pattern, age and sex of individuals affected, and prevalence of the disorders. Therefore, we limited our analyses to DBMD. Because we estimated the point prevalence of DBMD, we only included individuals with DBMD who were alive on July 1, 2010, leaving a total of 720 cases.

To determine if the variability in site-specific prevalence was within expected random variation, controlling for site population demographics and surveillance procedures, we constructed a dataset with one record for each age-race/ethnicity-site stratum, with a total of 96 strata. The dataset variables were number of DBMD cases, total population, age category (5-year intervals as shown in Table 2), surveillance site, race/ethnicity (White, Black, Hispanic and Other, which included Asian, Pacific Islander, American Indian, and unknown or unspecified race), method of diagnosis (proxy for diagnostic certainty; defined as genetic diagnosis in case or family member, family history of MD, or clinical diagnosis), the average number of reporting sources per patient (proxy for likelihood of identification at surveilled facilities), and the proportion of patients within the stratum treated at a MD clinic (proxy for likelihood of being treated at surveilled facilities). Data were too sparse to include zip code in the strata definition, which would have allowed us to use Census data as a proxy for socioeconomic status. We defined age and vital status as of July 1, 2010.

### Sources of variation in calculated prevalence

We calculated the unadjusted prevalence of DBMD overall and by site, age, and race/ethnicity. We calculated standardized prevalence for the US population using standard methods [15]. Briefly, we analyzed the prevalence for each age-race/ethnicity stratum, calculated the expected number of cases for the US based on the US population for equivalently-defined strata, then assessed the prevalence using the projected number of cases. Similar methods were used for standardized prevalence for subpopulations. We used the July 1, 2010 US Census estimated population of the surveillance catchment areas and the United States for all prevalence calculations and statistical models.

We used our theoretical model to develop a multivariable Poisson regression model to quantify the contribution of each measured source of variation to the total variance and how much variation remained unexplained. The Poisson model, fit to the stratum level dataset, controlled for the potential sources of uncertainty for which we had data. The MD STAR*net* data did not include a measure of socioeconomic status. Independent variables were age group, race/ethnicity, method of diagnosis, average number of reporting sources per patient, and whether the patient was treated at a specialized neuromuscular clinic. The natural log of the total stratum population was used as an offset variable to adjust for the differences in opportunity for the outcome. The number of DBMD cases in each stratum was the dependent variable. Analysis of deviance, the difference between the predicted outcome variables and the actual values for each record, was used to quantify the contribution of each variable to the variation in prevalence among the 96 strata.

We compared the unadjusted, standardized and modeled estimates of prevalence to assess the extent to which controlling for age, race/ethnicity and differences in surveillance process explained prevalence differences between sites. Primary analyses were conducted in R software, version 3.4.3 [16]. The secondary analyst used R software, version 3.6.0 [17] and SAS/STAT software, version 9.4 [18].

## Supplementary Information

**Additional file 1: Figure S2.** Disposition of articles from literature review.

**Additional file 2.** Articles included in full text review.

**Additional file 3: Table S1.** Investigators' estimates of bias, by source.

**Additional file 4.** Uncertainty in surveillance literature review search strategy.

**Additional file 5: Figure S1.** Survey of MD STARnet Investigators.

## Data Availability

Because of state policies governing access to public health surveillance data, MD STAR*net* data is only available through collaboration with a MD STAR*net* principal investigator. For more information on access to MD STAR*net* data, please contact the Centers for Disease Control and Prevention at mdstarnet@cdc.gov.
