# Supplementary material for: Sources of variation in estimates of Duchenne and Becker muscular dystrophy prevalence in the United States
Source: Orphanet J Rare Dis. 2023 Mar 22;18:65. doi: 10.1186/s13023-023-02662-0 (PMC10031951; doi:10.1186/s13023-023-02662-0)
Supplement: Supplementary file 1 — Additional file 1: Figure S2. Disposition of articles from literature review. [file 13023_2023_2662_MOESM1_ESM.docx]

Additional file 1: Figure S2. Disposition of Articles from Literature Review
