# Supplementary material for: Sources of variation in estimates of Duchenne and Becker muscular dystrophy prevalence in the United States
Source: Orphanet J Rare Dis. 2023 Mar 22;18:65. doi: 10.1186/s13023-023-02662-0 (PMC10031951; doi:10.1186/s13023-023-02662-0)
Supplement: Supplementary file 2 — Additional file 2. Articles included in full text review. [file 13023_2023_2662_MOESM2_ESM.docx]

**Additional file 2. Articles Included in Full Text Review**

We list below the articles that underwent full text review and indicate which articles were abstracted.

1. Besag J, Newell J. The detection of clusters in rare disease. Journal of the Royal Statistical Society Series A (Statistics in Society). 1991;154(1):143-55. [Included]

2. Edlin BR, Eckhardt BJ, Shu MA, Holmberg SD, Swan T. Toward a more accurate estimate of the prevalence of hepatitis C in the United States. Hepatology (Baltimore, Md). 2015;62(5):1353-63.

3. Hollak CE, Aerts JM, Ayme S, Manuel J. Limitations of drug registries to evaluate orphan medicinal products for the treatment of lysosomal storage disorders. Orphanet J Rare Dis. 2011;6:16. [Included]

4. Leadley RM, Lang S, Misso K, Bekkering T, Ross J, Akiyama T, et al. A systematic review of the prevalence of Morquio A syndrome: challenges for study reporting in rare diseases. Orphanet J Rare Dis. 2014;9:173.

5. Marsh K, Mahy M, Salomon JA, Hogan DR. Assessing and adjusting for differences between HIV prevalence estimates derived from national population-based surveys and antenatal care surveillance, with applications for Spectrum 2013. AIDS. 2014;28 Suppl 4:S497-505.

6. Mendez EP, Lipton R, Ramsey-Goldman R, Roettcher P, Bowyer S, Dyer A, et al. US incidence of juvenile dermatomyositis, 1995-1998: results from the National Institute of Arthritis and Musculoskeletal and Skin Diseases Registry. Arthritis Rheum. 2003;49(3):300-5. [Included]

7. Papoz L, Balkau B, Lellouch J. Case counting in epidemiology: limitations of methods based on multiple data sources. Int J Epidemiol. 1996;25(3):474-8. [Included]

8. Robertson C, Nelson TA, MacNab YC, Lawson AB. Review of methods for space-time disease surveillance. Spatial and spatio-temporal epidemiology. 2010;1(2-3):105-16.

9. Romanelli AM, Raciti M, Protti MA, Prediletto R, Fornai E, Faustini A. How Reliable Are Current Data for Assessing the Actual Prevalence of Chronic Obstructive Pulmonary Disease? PLoS One. 2016;11(2):e0149302.

10. Ward ZJ, Long MW, Resch SC, Gortmaker SL, Cradock AL, Giles C, et al. Redrawing the US Obesity Landscape: Bias-Corrected Estimates of State-Specific Adult Obesity Prevalence. PLoS One. 2016;11(3):e0150735.

11. Yu JB, Gross CP, Wilson LD, Smith BD. NCI SEER public-use data: applications and limitations in oncology research. Oncology (Williston Park). 2009;23(3):288-95. [Included]

12. Zu Erbach-Schoenberg E, Alegana VA, Sorichetta A, Linard C, Lourenco C, Ruktanonchai NW, et al. Dynamic denominators: the impact of seasonally varying population numbers on disease incidence estimates. Popul Health Metr. 2016;14:35.
