# Supplementary material for: Sources of variation in estimates of Duchenne and Becker muscular dystrophy prevalence in the United States
Source: Orphanet J Rare Dis. 2023 Mar 22;18:65. doi: 10.1186/s13023-023-02662-0 (PMC10031951; doi:10.1186/s13023-023-02662-0)
Supplement: Supplementary file 3 — Additional file 3: Table S1. Investigators' estimates of bias, by source. [file 13023_2023_2662_MOESM3_ESM.docx]

**Additional file 3: Table S1. Investigators Estimates of Bias, By Source**

|  | **< 5%** | **5-9%** | **10-19%** | **20-30%** | **30-49%** | **≥50%** | **Average^1^** |
| --- | --- | --- | --- | --- | --- | --- | --- |
|  | **Count** | **Count** | **Count** | **Count** | **Count** | **Count** | **Percent** |
| **Undiagnosed cases of DMD** | 3 | 0 | 4 | 0 | 0 | 0 | 9.4 |
| **Unascertained cases of DMD that meet the case definition** | 2 | 3 | 1 | 0 | 0 | 0 | 6.8 |
| **True DMD cases classified as possible cases due to missing or incorrect information** | 2 | 2 | 2 | 0 | 0 | 0 | 8.0 |
| **True DMD cases excluded due to missing or incorrect information** | 3 | 2 | 1 | 0 | 0 | 0 | 6.0 |
| **DMD cases that reside in the study area but obtain care from providers outside of the surveillance area** | 2 | 3 | 0 | 0 | 0 | 0 | 5.2 |
| **DMD cases who move into or out of the surveillance area** | 2 | 3 | 0 | 1 | 0 | 0 | 8.5 |
| **Demographic changes in the surveillance population** | 2 | 3 | 0 | 0 | 0 | 0 | 5.2 |
| **Difference between MD STARnet surveillance population and US population** | 2 | 2 | 0 | 0 | 1 | 0 | 11.7 |

^1^ Calculated as the midpoint of the category range times the count for the category divided by the total number of responses.
