# Supplementary material for: Sources of variation in estimates of Duchenne and Becker muscular dystrophy prevalence in the United States
Source: Orphanet J Rare Dis. 2023 Mar 22;18:65. doi: 10.1186/s13023-023-02662-0 (PMC10031951; doi:10.1186/s13023-023-02662-0)
Supplement: Supplementary file 4 — Additional file 4. Uncertainty in surveillance literature review search strategy. [file 13023_2023_2662_MOESM4_ESM.docx]

**Additional file 4. Uncertainty in Surveillance Literature Review Search Strategy**

Below are the search terms and MeSH terms used for the four PubMed searches for the literature search. The Google Scholar search did not allow for complex search terminology. Therefore, we conducted multiple searches using combinations of the terms below.

**underestimat* prevalence surveillance rare**

(underestimation [All Fields] OR underestimate[All Fields] OR underestimate [All Fields] OR underestimated[All Fields] OR underestimated'[All Fields] OR underestimated because[All Fields] OR underestimation[All Fields] OR underestimated [All Fields] OR underestimates[All Fields] OR underestimates [All Fields] OR underestimates [All Fields] OR underestimating[All Fields] OR underestimation[All Fields] OR underestimation'[All Fields] OR underestimations[All Fields] OR underestimations[All Fields] OR underestimator [All Fields] OR underestimator [All Fields] OR underestimators [All Fields] OR underestimators [All Fields]) AND (("epidemiology"[Subheading] OR "epidemiology"[All Fields] OR "prevalence"[All Fields] OR "prevalence"[MeSH Terms]) AND ("epidemiology"[Subheading] OR "epidemiology"[All Fields] OR "surveillance"[All Fields] OR "epidemiology"[MeSH Terms] OR "surveillance"[All Fields]) AND rare[All Fields]) AND ("loattrfree full text"[sb] AND "2006/11/07"[PDat] : "2016/11/03"[PDat] AND "humans"[MeSH Terms])

**underestimat* estimat* prevalence surveillance**

(underestimation [All Fields] OR underestimate[All Fields] OR underestimate'[All Fields] OR underestimated[All Fields] OR underestimated'[All Fields] OR underestimated because[All Fields] OR underestimation[All Fields] OR underestimates [All Fields] OR underestimating[All Fields] OR underestimation[All Fields] OR underestimation'[All Fields] OR underestimations[All Fields] OR underestimations[All Fields] OR underestimator[All Fields] OR underestimators [All Fields] AND (estimat[All Fields] OR estimata[All Fields] OR estimatability[All Fields] OR estimable All Fields] OR estimated[All Fields] OR estimation[All Fields] OR estimated [All Fields] OR estimate[All Fields] OR estimates[All Fields] OR estimated approximately[All Fields] OR estimated[All Fields] OR estimated by[All Fields] OR estimated indirectly[All Fields] OR estimated positively[All Fields] OR estimated the[All Fields] OR estimated to[All Fields] OR estimated total[All Fields] OR estimater[All Fields] OR estimate full[All Fields] OR estimating[All Fields] OR estimation[All Fields] OR estimator[All Fields] OR estimate project[All Fields] OR estimater[All Fields] OR estimaters [All Fields] OR estimates[All Fields] OR estimating[All Fields] estimation[All Fields] OR estimation'[All Fields] OR estimations[All Fields] OR estimation methods[All Fields] AND (("epidemiology"[Subheading] OR "epidemiology"[All Fields] OR "prevalence"[All Fields] OR "prevalence"[MeSH Terms]) AND ("epidemiology"[Subheading] OR "epidemiology"[All Fields] OR "surveillance"[All Fields] OR "epidemiology"[MeSH Terms] OR "surveillance"[All Fields])) AND ("loattrfree full text"[sb] AND "2006/11/07"[PDat] : "2016/11/03"[PDat] AND "humans"[MeSH Terms] AND English[lang])

**uncertainty in surveillance systems**

(("uncertainty"[MeSH Terms] OR "uncertainty"[All Fields]) AND ("epidemiology"[Subheading] OR "epidemiology"[All Fields] OR "surveillance"[All Fields] OR "epidemiology"[MeSH Terms] OR "surveillance"[All Fields]) AND systems[All Fields]) AND ("loattrfull text"[sb] AND "2006/11/07"[PDat] : "2016/11/03"[PDat] AND English[lang])

**surveillance prevalence uncertain* underestimat* estimat***

((("epidemiology"[Subheading] OR "epidemiology"[All Fields] OR "surveillance"[All Fields] OR "epidemiology"[MeSH Terms] OR "surveillance"[All Fields]) AND ("epidemiology"[Subheading] OR "epidemiology"[All Fields] OR "prevalence"[All Fields] OR "prevalence"[MeSH Terms])) AND (uncertain[All Fields] OR uncertain'[All Fields] OR uncertainties[All Fields] OR uncertainty[All Fields] OR uncertain[All Fields] uncertainess[All Fields] OR uncertainties[All Fields] OR uncertainty[All Fields] OR uncertainly[All Fields] OR uncertainness[All Fields] OR uncertainties that[All Fields] AND (underestimation[All Fields] OR underestimate[All Fields] OR underestimate'[All Fields] OR underestimated[All Fields] OR underestimated'[All Fields] OR underestimated because[All Fields] OR underestimation[All Fields] OR underestimaters[All Fields] OR underestimates[All Fields] OR underestimating[All Fields] OR underestimation[All Fields] OR underestimator[All Fields] OR underestimators All Fields] OR underestimators [All Fields]) AND (estimat[All Fields] OR estimata[All Fields] OR estimatability[All Fields] OR estimatable[All Fields] OR estimated[All Fields] OR estimation[All Fields] estimatecentral[All Fields] OR estimated[All Fields] OR estimated'[All Fields] OR estimated1[All Fields] OR estimated14[All Fields] OR estimated2[All Fields] OR estimated48[All Fields] OR estimated approximately[All Fields] OR estimated[All Fields] OR estimated by[All Fields] OR estimated indirectly[All Fields] OR estimated positively[All Fields] OR estimated[All Fields] OR estimated the[All Fields] OR estimated to[All Fields] OR estimated total[All Fields] OR estimater[All Fields] OR estimate full[All Fields] OR estimate in[All Fields] OR estimation[All Fields] OR estimate project[All Fields] OR estimater[All Fields] OR estimaters[All Fields] OR estimates[All Fields] OR estimates'[All Fields] OR estimates''[All Fields] OR estimating [All Fields] estimatinon[All Fields] OR estimation[All Fields] OR estimations[All Fields] OR estimator [All Fields] AND ("loattrfull text"[sb] AND "2006/11/07"[PDat] : "2016/11/03"[PDat] AND English[lang])
