# Supplementary material for: Sources of variation in estimates of Duchenne and Becker muscular dystrophy prevalence in the United States
Source: Orphanet J Rare Dis. 2023 Mar 22;18:65. doi: 10.1186/s13023-023-02662-0 (PMC10031951; doi:10.1186/s13023-023-02662-0)
Supplement: Supplementary file 5 — Additional file 5: Figure S1. Survey of MD STARnet Investigators. [file 13023_2023_2662_MOESM5_ESM.docx]

**Additional file 5: Figure S1. Survey of MD STARnet Investigators**

**MD STARnet SOURCES OF UNCERTAINTY**

**SURVEY OF INVESTIGATORS AND ANALYSTS**

This survey explores potential sources of uncertainty in MD STARnet data and in the extrapolation of prevalence estimates based on those data to national prevalence estimates. Please share the survey with any members of your MD STARnet team you think appropriate.

Below you will find a set of questions for each type of muscular dystrophy ascertained by MD STARnet. In responding to the questions, please consider the **expanded pilot MD STARnet data** compared to a **perfectly accurate measure** of the number of muscular dystrophy cases in a given geographic area during a given time period.

Please estimate the amount of potential bias introduced by each source of uncertainty. If you feel unable to estimate the amount of bias from each source, please rank the sources by their relative contribution to the uncertainty of the estimates, with 1 the source that contributes the most uncertainty.

## Duchenne Muscular Dystrophy (DMD)

| **Source of uncertainty** | **% bias in estimate** | | | | | |
| --- | --- | --- | --- | --- | --- | --- |
|  | < 5% | 5-9 | 10-19 | 20-30 | 30-49 | ≥50 |
| Undiagnosed cases of DMD |  |  |  |  |  |  |
| Unascertained cases of DMD that meet the case definition |  |  |  |  |  |  |
| True DMD cases classified as possible cases due to missing or incorrect information |  |  |  |  |  |  |
| True DMD cases excluded due to missing or incorrect information |  |  |  |  |  |  |
| DMD cases that reside in the study area but obtain care from providers outside of the surveillance area |  |  |  |  |  |  |
| DMD cases who move into or out of the surveillance area |  |  |  |  |  |  |
| Demographic changes in the surveillance population |  |  |  |  |  |  |
| True difference between MD STARnet surveillance population and US population |  |  |  |  |  |  |

Please provide describe any additional sources of uncertainty you think affects the use of the MD STARnet data for US DMD prevalence estimates.

___________________________________________________________________________________________________________________________________________________________________________________________________________________________________________________
